# Changes in soil microbial community composition and organic carbon fractions in an integrated rice–crayfish farming system in subtropical China

**DOI:** 10.1038/s41598-017-02984-7

**Published:** 2017-06-06

**Authors:** Guohan Si, Chenglin Peng, Jiafu Yuan, Xiangyu Xu, Shujun Zhao, Dabing Xu, Jinshui Wu

**Affiliations:** 1 College of Resources and Environment, Huazhong Agricultural University, Wuhan, 430064 China; 2 Qianjiang Scientific Observing and Experimental Station of Agro-Environment and Arable Land Conservation, Institute of Plant Protection and Soil Fertilizer, Hubei Academy of Agricultural Sciences, Wuhan, 430064 China; 3 Key Laboratory of Agro-ecological Processes in Subtropical Region, Institute of Subtropical Agriculture, Chinese Academy of Sciences, Changsha, 410125 China

## Abstract

Integrated rice**–**crayfish farming system is a highly efficient artificial ecosystem in which the rice (*Oryza sativa*) variety ‘Jianzhen 2′ is cultivated in waterlogged paddy fields along with crayfish (*Procambarus clarkii*). We investigated soil carbon fractions and microbial community structure by phospholipid fatty acids (PLFA) analysis in a 10-year field experiment using an integrated rice**–**crayfish (CR) model and a rice monoculture (MR) model at soil depths of 0–10 cm, 10–20 cm, 20–30 cm, and 30–40 cm. Compared with the MR model, the CR model had significantly more total organic carbon, particulate organic carbon, and dissolved organic carbon contents in all of the layers examined and microbial biomass carbon content in the 20–40 cm layer. Principal components analysis revealed that microbial community composition in the CR model differed from that in the MR model in the 20–30 cm layer. Higher proportions of gram–negative bacteria, aerobic bacteria and fungi in the 20–30 cm soil layer were observed for the CR model than the MR model. These results indicate that the CR model increases soil carbon levels, and strongly affects microbial community composition and structure in the deeper layers of soil, thereby accelerating subsurface soil nutrient cycling.

## Introduction

Waterlogged paddy fields are formed when the groundwater level rises due to local topographical and hydrological conditions, leading to extended waterlogging or poor drainage^1^. Because of the presence of a high water table, many cultivated fields are gleyed to a certain extent. Waterlogged paddy soils cover about 4 × 10^6^ ha in China, and are one the most important low-yield soil types in subtropical China^2^. These waterlogged paddy fields occur mainly in lakeshore region, coastal region, mountain valleys, and polders of plains along rivers. Because of water coverage or waterlogged conditions, waterlogged paddies have relatively low soil temperatures, weak air circulation, slow organic matter decomposition, and accumulate soil-reducing substances^1, 3^. This combination results in imbalances in water, fertiliser, air, and thermal conditions in the soil, which seriously hinder crop growth and decrease productivity.

The double-cropping of crayfish (*Procambarus clarkii*) and rice (*Oryza sativa*) has been practiced in Louisiana, USA for several decades^4–7^. Unfortunately, damage to agricultural fields caused by crayfish has made this otherwise valuable resource a pest in many countries, such as Portugal and the USA^8, 9^. Direct adverse effects of crayfish on rice seeds and seedlings include uprooting, plant fragmentation^10^, seedling consumption^11^, and interference with seed germination and seedling establishment by increasing water turbidity^12–14^. Indirect adverse effects include damage caused by the burrowing activity of crayfish, which results in the destruction of levees and loss of water from rice fields, causing significant losses in rice yields^15, 16^.

In the integrated rice**–**crayfish (CR) cultivation model, rice is cultivated in waterlogged paddy fields along with crayfish. Because of the high water table of waterlogged paddy fields, there is less water loss from such paddy fields caused by crayfish burrowing. A peripheral trench is excavated in the CR model that provides a refuge for crayfish during field puddling, seedling planting, and field drying, and prevents the negative effects of crayfish bioturbation on rice seedlings. Crayfish re-enter paddy fields after re-watering, when the rice stems are too strong to be destroyed by the crayfish. The CR model has become the primary cultivation model in waterlogged areas in subtropical China, and is performed in approximately 1.4 × 10^5^ ha in Hubei Province, China. The CR model fully utilises the shallow water environment and winter fallow period of rice paddies, and provides the maximum benefit by using the energy and materials available. Compared to the rice monoculture (MR) model, the CR model can help to alleviate resource scarcity, improve the food supply, and increase farmers’ incomes^17, 18^. Si *et al*.^19^ reported that the average productivity of the CR model is 40188 RMB·hm^−2^ higher than that of the MR model, confirming that it confers socioeconomic benefits.

Crayfish dig burrows as refugia against predation, dehydration, and environmental stress, as well as to nest^16^. Moreover, crayfish do not seem to return to their previously occupied burrows at the end of either their wandering phases or their foraging excursions^20^. Burrows of various depths are usually directed downwards to the water table and terminate in an enlarged chamber with water at the bottom^16^. The depth of most burrows is dependent on the groundwater level, which is usually 50–80 cm. Crayfish burrowing activity disturbs the surface and base layers of waterlogged paddy soils, which increases soil permeability and affects air and water circulation and plant nutrition, thereby mediating soil chemical and biological processes. Studies on crayfish in rice fields have primarily focused on damage to rice crops^10, 21, 22^, water loss from rice fields, and the collapse of delimiting banks caused by burrowing crayfish^23, 24^. To our knowledge, no study has investigated how waterlogged soil’s permeability changes after crayfish burrowing activity, and how this affects the chemical and biological processes in the soil. The aim of this study was to determine the effects of the CR model on soil organic carbon, enzyme activity, and microbial community structure in a waterlogged paddy soil. The results could be used to support further research into the processes involved in the environmental changes caused by the CR model, as well as providing a theoretical basis for promoting the model.

## Results

### Soil physicochemical properties

As shown in Table 1, the total nitrogen (N) and phosphorus (P) contents decreased with increasing soil depth in both models, and pH and bulk density increased with increasing soil depth. The total potassium (K) content and carbon: nitrogen (C:N) ratio in the CR model increased with increasing soil depth. The total K contents of the 0–10 cm, 10–20 cm, 20–30 cm, and 30–40 cm layers were significantly higher in the CR model than in the MR model by 5.1%, 5.0%, 8.4%, and 10.1%, respectively. The total N contents of the 0–10 cm, 10–20 cm, and 20–30 cm layers were significantly higher in the CR model than in the MR model by 29.9%, 23.0%, and 28.7%, respectively. There were no significant differences in the total P content at a depth of 10–40 cm between the CR and MR models, but the total P content in the 0–10 cm layer was significantly higher (9.8%) in the CR model than in the MR model. Bulk density was considerably lower in the CR model than in the MR model in all of the layers examined. The pH values and C:N ratio showed an increasing trend in the CR model compared with the MR model in all of the layers examined, but the differences were not statistically significant (*p* > 0.05).

**Table 1:** Soil physicochemical properties under different treatments.

Soil depths	pH		Bulk density (g·cm^−3^)		Total N (g·kg^−1^)		Total P (g·kg^−1^)		Total K (g·kg^−1^)		C:N ratio	
(cm)	MR	CR	MR	CR	MR	CR	MR	CR	MR	CR	MR	CR
0–10	7.17 ± 0.13a	7.29 ± 0.04a	1.09 ± 0.03a	0.90 ± 0.01b	1.94 ± 0.22b	2.52 ± 0.28a	0.41 ± 0.01b	0.45 ± 0.02a	17.30 ± 0.25b	18.17 ± 0.09a	8.09 ± 0.18a	8.35 ± 0.69a
10–20	7.20 ± 0.08a	7.34 ± 0.04a	1.36 ± 0.02a	1.10 ± 0.05b	1.70 ± 0.13b	2.09 ± 0.19a	0.40 ± 0.02a	0.43 ± 0.01a	17.60 ± 0.19b	18.48 ± 0.15a	8.34 ± 0.32a	8.32 ± 1.05a
20–30	7.21 ± 0.11a	7.38 ± 0.06a	1.29 ± 0.03a	1.18 ± 0.11b	1.44 ± 0.12b	1.85 ± 0.18a	0.38 ± 0.02a	0.37 ± 0.05a	17.11 ± 0.34b	18.56 ± 0.20a	7.98 ± 0.37a	8.47 ± 0.52a
30–40	7.31 ± 0.11a	7.45 ± 0.05a	1.28 ± 0.02a	1.16 ± 0.01b	0.94 ± 0.07a	1.13 ± 0.12a	0.34 ± 0.03a	0.34 ± 0.04a	17.27 ± 0.06b	19.01 ± 0.06a	8.17 ± 0.47a	9.02 ± 0.35a

MR, rice monoculture model; CR, Integrated rice–crayfish model. Means with different letters for the same property in the same soil layer indicate significant differences at *p* < 0.05. Values are means ± standard errors.

### Soil enzyme activity

Soil enzyme activity decreased with increasing soil depth (Table 2). Soil invertase and acid phosphatase activities showed a decreasing trend in CR model compared with the MR model in all of the layers examined, but the differences were not statistically significant (*p* > 0.05). Soil urease activity showed a decreasing trend in the CR model compared with the MR model at a depth of 0–30 cm, and the difference was significant in the 10–20 cm layer.

**Table 2:** Soil enzyme activity under different treatments.

Soil depths	Invertase (mg glucose·g^−1^ soil·24 h^−1^)		Urease (mg NH_3_-N·g^−1^ soil·24 h^−1^)		Acid phosphatase (mg phenol·g^−1^ soil·2 h^−1^)	
(cm)	MR	CR	MR	CR	MR	CR
0–10	24.29 ± 2.17a	21.84 ± 2.13a	0.90 ± 0.02a	0.83 ± 0.06a	0.52 ± 0.04a	0.45 ± 0.02a
10–20	20.62 ± 2.72a	16.28 ± 2.19a	0.66 ± 0.06a	0.55 ± 0.01b	0.39 ± 0.03a	0.35 ± 0.03a
20–30	17.51 ± 3.37a	14.38 ± 0.77a	0.49 ± 0.03a	0.46 ± 0.08a	0.34 ± 0.03a	0.33 ± 0.04a
30–40	7.42 ± 0.87a	7.18 ± 1.31a	0.23 ± 0.01a	0.27 ± 0.02a	0.14 ± 0.02a	0.13 ± 0.02a

MR, rice monoculture model; CR, Integrated rice–crayfish model. Means with different letters for the same property in the same soil layer indicate significant differences at *p* < 0.05. Values are means ± standard errors.

### Soil organic carbon fractions

Total organic carbon (TOC), particulate organic carbon (POC) and microbial biomass carbon (MBC) contents decreased with increasing soil depth, but dissolved organic carbon (DOC) content changed little (Table 3). The TOC, POC, and DOC contents were significantly greater in the CR model than in the MR model in all of the layers examined. In the 0–10 cm, 10–20 cm, 20–30 cm, and 30–40 cm layers, the TOC contents in the CR model were greater than those in the MR model by 33.5%, 22.6%, 36.7%, and 31.6%, respectively, and the DOC contents were 58.4%, 73.2%, 72.4%, and 32.7% greater, respectively. The MBC contents of the 20–30 cm and 30–40 cm layers were significantly greater in the CR model than in the MR model by 34.1% and 40.0%, respectively. The POC contents in the 0–10 cm, 10–20 cm, 20–30 cm, and 30–40 cm layers in the CR model were greater than those in the MR model by 36.8%, 56.1%, 59.7%, and 50.0%, respectively.

**Table 3:** Soil organic carbon and its active components under different treatments.

Soil depths	TOC (g·kg^−1^)		MBC (mg·kg^−1^)		DOC (mg·kg^−1^)		POC (g·kg^−1^)	
(cm)	MR	CR	MR	CR	MR	CR	MR	CR
0–10	15.66 ± 1.48b	20.90 ± 0.81a	307.43 ± 28.30a	294.37 ± 43.10a	30.73 ± 4.80b	48.67 ± 4.02a	4.56 ± 0.05b	6.24 ± 0.72a
10–20	14.15 ± 0.86b	17.34 ± 1.17a	216.85 ± 11.35a	260.62 ± 34.83a	27.94 ± 2.21b	48.38 ± 3.09a	3.19 ± 0.54b	4.98 ± 0.87a
20–30	11.50 ± 1.28b	15.73 ± 1.73a	195.18 ± 16.59b	261.69 ± 26.50a	29.35 ± 1.24b	50.31 ± 7.09a	2.95 ± 0.19b	4.71 ± 0.51a
30–40	7.72 ± 0.87b	10.16 ± 0.99a	97.07 ± 10.44b	135.93 ± 17.19a	30.22 ± 2.24b	40.10 ± 4.87a	1.16 ± 0.12b	1.74 ± 0.28a

MR, rice monoculture model; CR, Integrated rice–crayfish model. TOC, total organic carbon; MBC, microbial biomass carbon; DOC, dissolved organic carbon; POC, particulate organic carbon. Means with different letters for the same property in the same soil layer indicate significant differences at *p* < 0.05. Values are means ± standard errors.

### Soil microbial community structure

**Figure 1:**
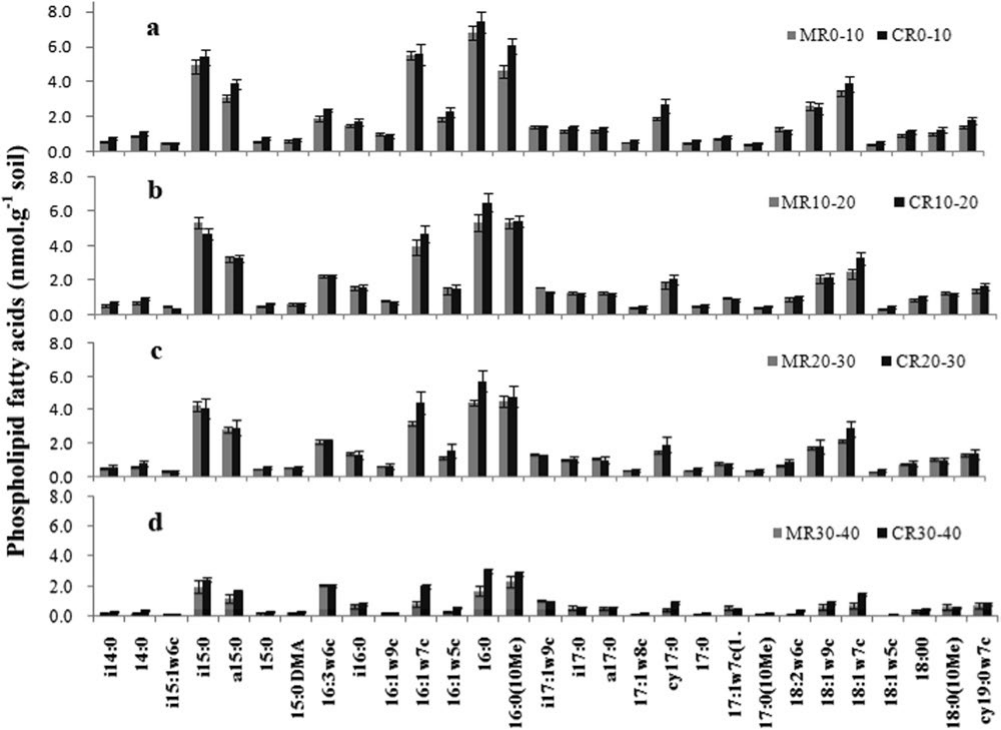
Phospholipid fatty acids profiles in 0–10 cm soil layer (**a**), 10–20 cm soil layer (**b**), 20–30 cm soil layer (**c**), and 30–40 cm soil layer (**d**). CR 0–10, CR 10–20, CR 20–30, and CR 30–40 indicate 0–10 cm, 10–20 cm, 20–30 cm, and 30–40 cm soil layers in the integrated rice–crayfish model; MR 0–10, MR 10–20, MR 20–30, and MR 30–40 indicate the soil layers in the rice monoculture model.

Twenty-nine phospholipid fatty acids (PLFA) with chain lengths of C14 to C19 were identified (Fig. 1). The total amount of PLFA expressed in nmol·g^−1^ dry soil is an indicator of the total microbial biomass present, and it decreased with soil depth in both models. As shown in Table 4, the total amount of total bacteria and fungi and the ratio of aerobic to anaerobic bacteria (aerobe/anaerobe), in the 30–40 cm layer were considerably higher in the CR model than in the MR model by 47.6%, 60.7%, and 54.2%, respectively. There were considerably more aerobic bacteria, gram–negative (G−) bacteria, and arbuscular mycorrhizal fungi (AMF) in the 20–30 cm layer in the CR model than in the MR model by 25.6%, 28.6%, and 43.2%, respectively, and by 85.9%, 83.9%, and 106.5%, respectively, in the 30–40 cm layer. The ratio of gram–positive (G+) to G− bacteria (G+/G−) was considerably lower in the CR model than in the MR model at a depth of 20–40 cm. In the 0–10 cm layer, there were considerably more actinomycetes in the CR model than in the MR model, but the ratio of fungal to bacterial PLFAs (F/B) was considerably lower in the CR model than in the MR model. There was no significant difference in the amount of G+ bacteria or anaerobic bacteria between the CR and MR models in any of the layers examined.

**Table 4:** Soil microbial community composition under different treatments.

Microbial community composition (nmol·g^−1^)	0–10 cm		10–20 cm		20–30 cm		30–40 cm	
	MR	CR	MR	CR	MR	CR	MR	CR
Total bacteria	24.84 ± 2.81a	28.75 ± 2.45a	23.45 ± 3.00a	24.62 ± 2.95a	19.71 ± 1.11a	22.12 ± 2.61a	8.20 ± 1.11b	12.07 ± 1.43a
G + bacteria	12.28 ± 1.82a	14.35 ± 0.92a	13.33 ± 1.35a	12.46 ± 1.83a	11.15 ± 1.31a	11.06 ± 0.91a	5.26 ± 0.55a	6.58 ± 0.99a
G− bacteria	15.84 ± 1.61a	17.94 ± 1.67a	12.80 ± 1.17a	14.71 ± 1.47a	10.62 ± 0.89b	13.66 ± 0.97a	3.60 ± 0.54b	6.62 ± 1.01a
Actinomycetes	6.01 ± 0.38b	7.76 ± 0.79a	7.02 ± 0.73a	7.07 ± 1.34a	6.00 ± 0.89a	6.12 ± 0.53a	3.19 ± 0.34a	3.67 ± 0.47a
Fungi	3.87 ± 0.32a	3.58 ± 0.37a	2.99 ± 0.23a	3.14 ± 0.37a	2.44 ± 0.23a	2.67 ± 0.34a	0.84 ± 0.13b	1.35 ± 0.13a
F/B	0.16 ± 0.02a	0.13 ± 0.00b	0.13 ± 0.01a	0.13 ± 0.01a	0.12 ± 0.00a	0.12 ± 0.01a	0.10 ± 0.00a	0.11 ± 0.01a
G+/G−	0.77 ± 0.07a	0.80 ± 0.09a	1.04 ± 0.06a	0.85 ± 0.08a	1.05 ± 0.09a	0.81 ± 0.07b	1.46 ± 0.11a	0.99 ± 0.06b
Aerobic bacteria	9.47 ± 1.17a	9.48 ± 0.91a	7.31 ± 1.32a	8.06 ± 1.53a	5.87 ± 0.53b	7.37 ± 0.61a	1.92 ± 0.62b	3.57 ± 0.58a
Anaerobic bacteria	17.35 ± 2.62a	20.88 ± 2.26 a	19.36 ± 1.99a	18.43 ± 3.74a	16.27 ± 2.13a	16.24 ± 1.80a	8.08 ± 0.97a	9.73 ± 1.53a
Aerobe/Anaerobe	0.55 ± 0.06a	0.45 ± 0.06a	0.38 ± 0.03a	0.43 ± 0.05a	0.36 ± 0.05a	0.44 ± 0.06a	0.24 ± 0.03b	0.37 ± 0.03a
AMF	1.86 ± 0.25a	2.23 ± 0.18a	1.49 ± 0.25a	1.47 ± 0.38a	1.11 ± 0.17b	1.59 ± 0.14a	0.31 ± 0.06b	0.64 ± 0.11a

MR, rice monoculture model; CR, Integrated rice–crayfish model. G + , gram–positive bacteria, G−, gram–negative bacteria, F/B, ratio of fungal to bacterial PLFAs; G+/G−, the ratio of gram–positive to gram–negative bacteria; AMF, arbuscular mycorrhizal fungi; Aerobe/Anaerobe, the ratio of aerobic to anaerobic bacteria. Means with different letters for the same property in the same soil layer indicate significant differences at *p* < 0.05. Values are means ± standard errors.

**Figure 2:**
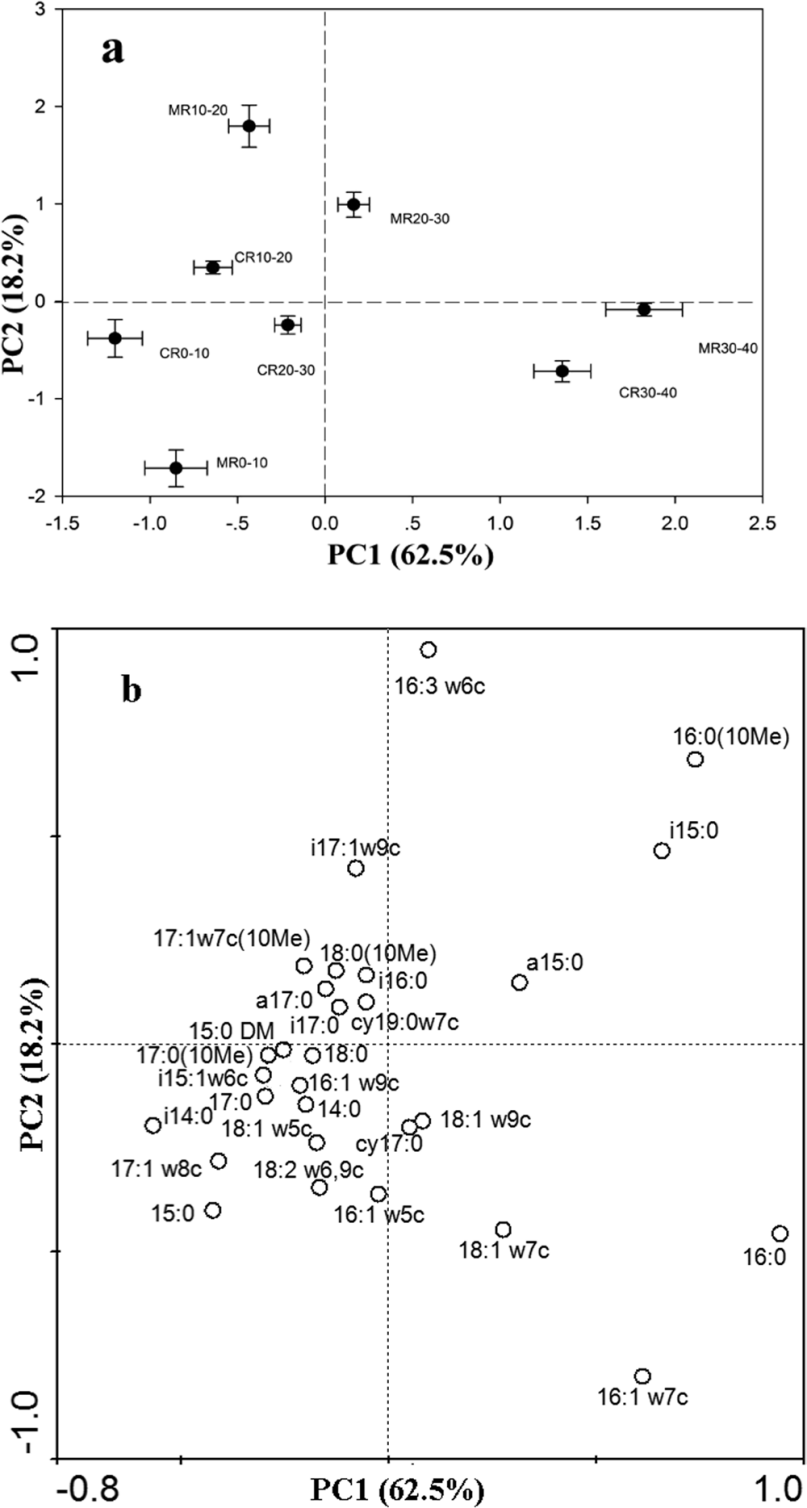
Principal components analysis of phospholipid fatty acid (PLFA) profiles (**a**) and loading values for individual PLFAs (**b**). CR 0–10, CR 10–20, CR 20–30, and CR 30–40 indicate 0–10 cm, 10–20 cm, 20–30 cm, and 30–40 cm soil layers in the integrated rice–crayfish model; MR 0–10, MR 10–20, MR 20–30, and MR 30–40 indicate the same in the rice monoculture model.

The principal components analysis assessed differences in microbial community structure between the CR and MR models in the four soil layers examined. The first principal component (PC1) accounted for 62.5% of the total variation, and the second component (PC2) for 18.2% of the variation (Fig. 2). The ordination plot in Fig. 2a illustrates the difference in PLFA composition between the two models in the different soil layers. There was a high degree of spatial variability in the vertical direction, with a strong separation based on soil depth. The PLFA profiles were significantly different between the CR and MR models in the 20–30 cm layer but not in the other layers, suggesting that bioturbation caused by the crayfish in the CR model altered the microbial community structure in the 20–30 cm soil layer.

Figure 2b shows the loading values for the individual PLFA biomarkers in the different models. For PLFAs from all the fractions, the proportions of most G+ bacterial PLFAs (a15:0, i15:0, 16:0, i17:0, and a17:0), anaerobic bacteria PLFAs (i15:0, a15:0, 10Me16:0, i17:0, a17:0, and 10Me18:0), and actinomycetes PLFAs (10Me16:0 and 10Me18:0) increased in the 20–30 cm layer in the MR model, while the proportions of most G− bacterial PLFAs (16:1ω5c, 16:1ω9c, 17:1ω8c, 18:1ω5c, 18:1ω9c and cy17:0), aerobic bacterial PLFAs (14:0, 15:1ω6c and 18:1ω9c), and fungal PLFAs (18:1ω9c and 18:2ω6,9c) increased in the 20–30 cm layer in the CR model. This indicates that compared to the MR model, the CR model contained higher proportions of G− bacteria, aerobic bacteria, and fungi in the 20–30 cm soil layer.

Correlations between soil physicochemical properties and microbial parameters are presented in Table 5. TOC, POC, and total N contents and enzyme activity were significantly positively correlated with the microbial parameters. MBC was significantly positively correlated with the microbial parameters, except for AMF. Total K and the C:N ratio exhibited nonsignificant correlations with the microbial parameters, and the DOC content was significantly positively correlated with the amount of total bacteria, G− bacteria, and actinomycetes. Bulk density was significantly positively correlated with the amount of total bacteria, G− bacteria, fungi, aerobic bacteria, and the ratio of aerobic to anaerobic bacteria. Total P was significantly positively correlated with the amount of total bacteria, G+ bacteria, G− bacteria, fungi, actinomycetes, and anaerobic bacteria. A significant positive correlation was found between pH and AMF.

**Table 5:** Pearson’s correlation analysis of microbial parameters and soil physicochemical properties.

	Total bacteria	G + bacteria	G− bacteria	Actinomycetes	Fungi	F/B	Aerobic bacteria	Anaerobic bacteria	Aerobic/Anaerobic bacteria	AMF
TOC	0.841**	0.789**	0.859**	0.783**	0.819**	0.456*	0.693**	0.671**	0.566**	0.518**
MBC	0.530**	0.516**	0.527**	0.491*	0.563**	0.427*	0.750**	0.629**	0.676**	NS
DOC	0.434*	NS	0.475*	0.422*	NS	NS	NS	NS	NS	NS
POC	0.786**	0.725**	0.818**	0.703**	0.784**	0.491*	0.598**	0.549**	0.537**	0.506*
pH	NS	NS	NS	NS	NS	NS	NS	NS	NS	−0.655**
Total N	0.843**	0.790**	0.864**	0.773**	0.839**	0.503*	0.718**	0.670**	0.624**	0.561**
Total P	0.779**	0.793**	0.737**	0.805**	0.718**	NS	NS	0.413*	NS	NS
Total K	NS	NS	NS	NS	NS	NS	NS	NS	NS	NS
C:N ratio	NS	NS	NS	NS	NS	NS	NS	NS	NS	NS
Bulk density	−0.431*	NS	−0.514*	NS	−0.433*	NS	−0.549**	NS	−0.516**	NS
Invertase	0.807**	0.810**	0.793**	0.746**	0.862**	0.629**	0.512*	0.492*	0.474*	0.768**
Urease	0.718**	0.691**	0.734**	0.602**	0.807**	0.667**	0.698**	0.595**	0.675**	0.742**
Acid phosphatase	0.794**	0.783**	0.789**	0.727**	0.860**	0.663**	0.645**	0.608**	0.588**	0.721**

NS: not significant; *p < 0.05; **p < 0.01.

## Discussion

In the present study, the bulk density was significantly lower in the CR model than in the MR model in all soil layers examined. Our results are in agreement with those of Wang *et al*.^25^, who reported that burrowing activity decreases bulk density. Crayfish feeding, molting, and excreting recycles nutrients, and increases the concentrations of elements such as N, P, and K. The total N content at a depth of 0–30 cm was significantly higher in the CR model than in the MR model. Crayfish penetrate the surface and base layers of rice paddy soils, which increases soil permeability. The long-term alternation of drying and wetting in a rice paddy makes soil K constantly move downward to deeper soil. The total K content in the CR model increased with increasing soil depth, and the total K contents of all of the soil layers examined were significantly higher in the CR model than in the MR model, supporting the findings of Bahmaniar *et al*.^26^. A possible reason for the increasing pH in the CR model compared with the MR model could be continuous winter waterlogging for crayfish cultivation, which increases the soil pH towards neutrality. The total P content in the 0–10 cm layer was significantly higher in the CR model than in the MR model (by 9.8%), but no significant differences in total P content at a depth of 10–40 cm were observed, possibly because most of the P additions were absorbed by the top soil and less P leached to the deeper layers. Soil enzymes have significant effects on soil moisture, aeration, and temperature, as do hydrological conditions, and soil moisture is an important ecological factor in rice wetland ecosystems^27^. Urease, acid phosphatase, and invertase are related to the N, P, and C cycles, respectively, and their activities show a decreasing trend in the CR model compared with the MR model in all of the soil layers examined; urease activity was significantly lower in the CR model than in the MR model in the 10–20 cm soil layer. Long-term flooding in the CR model can affect soil enzyme release by changing microbial community structure. Moreover, in reducing conditions, the concentrations of inhibitory factors, such as Fe^2+^, increase, thereby affecting soil enzyme activity^28^. Furthermore, in significantly anaerobic conditions, polyphenol oxidase activity is inhibited, leading to the accumulation of polyphenolic compounds. High concentrations of phenolic substances can inhibit the activity of hydrolytic enzymes that do not require oxygen, such as invertase, phosphatase, and urease^29^.

Soil organic carbon plays an essential role in improving soil quality and crop production by affecting soil chemical, physical, and biological properties^30^. In all of the soil layers examined, the TOC content in the CR model was significantly higher than that in the MR model. Inadequate amounts of oxygen under submerged conditions lead to even modest oxygen demands for microbial activity not being met, resulting in decreased rates of decomposition^31^. In the CR model, rice paddies are flooded during the winter fallow season for crayfish farming; consequently, organic matter degradation rate decreases. Moreover, uneaten crayfish feed, and the moult and excreta produced during the growth process supplement the soil. Therefore, with respect to no irrigation in the MR model during the winter fallow, the CR model was better at retaining organic carbon in the soil. The POC fraction, as a readily decomposable substrate for soil microorganisms, is sensitive to recent inputs of plant residue^32^. Significantly higher POC contents were found in the CR model than in the MR model in all the soil layers examined. Huryn *et al*.^33^ reported that the POC content is enriched by crayfish crushing and eating straw, leaves, and other plant litter. Moreover, soil bulk density decreases after crayfish burrowing activity, which thickens the plough layer and increases the biomass and rooting depth of rice roots. Large amounts of decaying dead roots greatly enrich POC in the soil. The MBC regulates soil organic matter decomposition and nutrient cycling, and thus plays a vital role in maintaining agroecosystem functioning and sustainability^34^. The MBC contents in the 20–30 cm and 30–40 cm layers were significantly higher in the CR model than in the MR model by 34.1% and 40.0%, respectively. This increase in soil microbial biomass may have been related to the improved ventilation caused by crayfish burrows, which allowed oxygen to reach deeper soil layers and thereby increased soil microbial biomass. The DOC is a useful indicator of soil quality and functioning, and reflects organic matter decomposition and nutrient release^35^. Significantly higher DOC contents were found in the CR model than in the MR model in all the soil layers examined. Flooded conditions can lead to the dispersion of aggregates and increase the dissolution of soil organic carbon^36^, thereby increasing the DOC content. Moreover, soil organic carbon is the main source of DOC^37^, and determines the soil’s DOC content.

Soil microbial communities play an important role in biogeochemical cycling, and as primary decomposers of soil organic matter^38^. Investigating microbial populations using PLFA analysis provides direct information for the identification, classification, and quantification of microbial community composition, and avoids the selectivity problems associated with culture techniques^39^. Burrowing activity significantly affects belowground processes by increasing the passage of liquid and gas between the soil and the environment^40, 41^. In the CR model, the high ratio of aerobic to anaerobic bacteria at a depth of 20–40 cm was probably caused by the accelerated population growth of aerobic bacteria. G− bacteria preferentially use fresh plant inputs as carbon sources, whereas G+ bacteria are thought to favour older and more microbially processed soil organic matter^42^. Fungi and G− bacteria quickly respond to organic carbon inputs in paddy soils^43^. Compared to the MR model, in the CR model, a significantly lower G+/G− ratio, more G− bacteria at a depth of 20–40 cm, and significantly more fungi in the 30–40 cm layer were observed. This may have been because the downward translocation of fresh litter detritus was mediated by crayfish burrowing activity, which increased G− bacterial and fungal growth. A higher F/B ratio is indicative of a sustainable agroecosystem with low environmental impact^44^. The F/B ratio in the 0–10 cm layer was significantly lower in the CR model than in the MR model, probably because the CR model remained waterlogging during the winter fallow, which increased anaerobic bacterial growth (see Table 4). There were significantly more AMF in the CR model than in the MR model at a depth of 20–40 cm, possibly because crayfish burrowing activity could increase soil porosity and decrease mechanical resistance to the growth of AM hyphae^45^. There were significantly more actinomycetes in the 0–10 cm layer in the CR model than in the MR model, which agrees with the results of Zhang *et al*.^46^, who reported that actinomycetes are more abundant in N-rich paddy soils. However, MacKenziem and Quideau^47^ reported that in forest and grassland soils, actinomycetes exhibit their highest activity when N is limiting. This discrepancy could be due to differences in crop species, soil properties, soil management, and their complex interactions^48^. The principal components analysis revealed that the crayfish affected the microbial community structure of the paddy soil, and that the CR model changed the microbial community structure in the 20–30 cm layer comparison to the MR model. In the CR model, crayfish penetrate the surface and base layers of the rice paddy soil, which increases soil permeability and water migration channels, and allows nutrients and oxygen to reach the base layer. These events ultimately affect microbial community structure in deeper soil layers.

The correlation analysis revealed that enzyme activity was significantly correlated with the soil microbial parameters as estimated by the PLFAs, supporting the results of Giacometti *et al*.^49^, who also reported strong interactions between microbial biomass and enzyme activity. Crayfish burrowing activity modifies the soil’s physical structure and decreases bulk density, which increases the potential for biological and chemical exchange by increasing the interface between soil and water/air. Bulk density was significantly positively correlated with the number of total bacteria, G− bacteria, fungi, aerobic bacteria, and the ratio of aerobic to anaerobic bacteria. P is an important factor in microbial community structure. Total P was significantly positively correlated with the amount of total bacteria, G+ bacteria, G− bacteria, fungi, actinomycetes, and anaerobic bacteria; however, the amount of AMF did not increase under a high phosphate concentration, which is inconsistent with the results of Beauregard *et al*.^50^. Our results are in agreement with those of Hackl *et al*.^51^, who reported that the PLFA 16:1ω5c, which is common in AMF, decreases with decreasing soil pH. TOC, POC, and MBC concentrations were significantly positively correlated with the microbial parameters, suggesting that high soil carbon concentrations increase microbial biomass in paddy soils. High soil organic matter levels result in the development of a large, active, microbial biomass that can decompose large amounts of newly available compounds^3^. DOC concentrations were significantly positively correlated with the amount of total bacteria, G− bacteria, and actinomycetes, supporting the results of Bossio *et al*.^52^, who reported that specific fatty acids that are common in actinomycetes and G− bacteria are associated with DOC concentrations.

In conclusion, compared with the MR model, the CR model had significantly more TOC, POC, and DOC contents in all the layers examined, and microbial biomass carbon content in the 20–40 cm layer. The amount of aerobic bacteria, AMF, and G− bacteria were more abundant at a depth of 20–40 cm in the MR model than in the CR model. The principal components analysis revealed differences in the microbial community composition between the CR and MR models in the 20–30 cm layer, and higher proportions of G− bacteria, aerobic bacteria, and fungi in the 20–30 cm soil layer in the CR model than in the MR model. Pearson’s correlation analysis showed that soil organic fractions, enzyme activity, total P, and bulk density were the key determinants of soil microbial community composition. Overall, the CR model could decrease bulk density, increase soil carbon levels, and strongly affect microbial community composition and structure in the deeper layers of soil, thereby accelerating subsurface soil nutrient cycling. Future studies should investigate the effects of fluctuating oxidising and reducing conditions, and the formation, storage, and turnover of soil organic matter on microbial community dynamics to elucidate the effects of crayfish bioturbation on soil microbial communities in waterlogged paddy soils.

## Materials and Methods

### Study area and experimental design

The study was conducted on a 10-year-old integrated rice–crayfish farming system in waterlogged paddy fields at Bailu Lake Farm, Qianjiang City, Hubei Province, China (30°11′36.07″N, 112°43′22.68″E). This region has a winter static groundwater level of 40–60 cm and a northern, humid, subtropical monsoon climate. The average annual temperature is 16.1 °C, with a frost-free period of 246 days. The average annual rainfall is 1100 mm. The soil type is a fluvo-aquic paddy soil, which developed from lake sediments.

An 1800-m^2^ field was uniformly divided into six 300-m^2^ plots, and three replicates were included for each treatment. The two treatments were as follows:

1. MR model: midseason rice monoculture model (fallow after the rice harvest, 1900 kg·ha^−1^ of straw returned to the field);
2. CR model: integrated rice–crayfish model (crayfish cultivation after the rice harvest, 1900 kg·ha^−1^ of straw returned to the field).

The field was puddled and leveled using a mouldboard plough in mid- or late June each year, and then flooded 2–3 days before planting. Thirty-five-day-old rice (Jianzhen 2) seedlings were planted on 20 June every year at a spacing of 16.7 cm × 26.6 cm, with two to three seedlings per hill. Chemical fertilisers were applied at a rate of 120 kg N·ha^−1^, 36.0 kg P_2_O_5_·ha^−1^, and 60.0 kg K_2_O·ha^−1^ per year.

A peripheral trench (3.0 m wide and 1.2 m deep) was excavated in the CR model that was used as a refuge for crayfish. The excavated soil was used to construct a 1.5-m-high dyke that was surrounded by a 0.3-m-high nylon net to prevent the crayfish from escaping. Crayfish larvae (weighing 5 ± 2 g) were stocked at a density of 1.5 × 10^5^ larvae·ha^−1^, and the crayfish self-propagated inside the rice paddies. At the same period in subsequent years, additional broodstock was added, as appropriate for the conditions. The crayfish were released into the flooded field on 25 October 2005, exactly 15 days after the rice harvest. Mature crayfish were harvested in the first 10 days of June, and immature crayfish migrated to the peripheral trench before re-entering the rice field after field puddling, seedling planting, field drying, and re-watering. In the second season, mature crayfish were harvested before the rice harvest. Supplementary feed for the crayfish was supplied daily from March to May (1.8 Mg·ha^−1^ per year). The N, P_2_O_5_, and K_2_O contents of the crayfish feed were 46.6 g·kg^−1^, 11.0 g·kg^−1^, and 10.5 g·kg^−1^, respectively. Under the CR model, a density of 0.4 burrows·m^−2^ was observed in the paddy fields.

### Soil sampling and storage

Soil samples were collected on the 10^th^ October 2015 after the harvest using a sample probe at depths of 0–10 cm, 10–20 cm, 20–30 cm, and 30–40 cm. Sampling was conducted from five different places within each replicate plot, and the five samples were mixed to prepare a composite sample for the plot. Immediately after sampling, visible root fragments and stones were manually removed, and the samples were mixed well and divided into two portions. One portion of fresh soil was passed through a 2-mm sieve and stored in a refrigerator at 4 °C until its biological characteristics were analysed, and the other was air-dried and filtered in preparation for chemical characteristics analysis. The moisture content of the individual samples was determined gravimetrically in 10-g portions after drying at 105 °C for 48 h.

### Soil physicochemical properties analysis

Soil pH was measured in a soil: water mixture (1:2.5 w/v) using a pH meter (UB-7, UltraBASIC, Denver, CO, USA), and bulk density was determined using a 5.0-cm-diameter core sampler. Total N was determined by the Kjeldahl digestion method. Total P and total K were extracted and determined by the perchloric acid digestion methods and spectrophotometer protocols^53^.

### Soil enzyme activity analysis

Enzyme activity was assayed according to the methods described by Guan^54^, and acid phosphatase activity was estimated by determining the amount of phenol released after incubating the samples with phenyl disodium phosphate (0.5% w/v) for 2 h at 37 °C. Urease activity was measured by determining the amount of NH_4_^+^ released from a hydrolysis reaction after incubating the samples with urea (10% w/v) for 24 h at 37 °C, and invertase activity was measured by determining the amount of glucose released after incubating the samples with sucrose (8% w/v) at 37 °C for 24 h.

### Soil organic carbon fractions analysis

The TOC content was determined by oxidation with potassium dichromate and titration with ferrous ammonium sulfate. The DOC was extracted as described by Jiang *et al*.^55^. Moist soil samples (10-g oven-dry weight) were shaken with 25 mL of distilled water for 1 h at 250 r·min^−1^, and then centrifuged for 10 min at 4000 × *g*. The supernatant was filtered through a 0.45-μm membrane filter. The DOC content of the filtrate was measured by oxidation with potassium dichromate and titration with ferrous ammonium sulfate. The POC content was determined using the method described by Cambardella and Elliott^56^; 20 g of air-dried soil (<2 mm) was dispersed in 100 mL of sodium hexametaphosphate (5 g·L^−1^) by shaking by hand for 15 min and then on a reciprocating shaker (180 r·min^−1^) for 18 h. The soil suspension was poured through a 53-mm sieve with distilled water. All of the material that remained on the sieve was particulate organic matter, and was washed into a dry dish, oven-dried at 60 °C, and weighed and ground to pass through a 15-mm sieve. The POC content was determined using the same method as described for the TOC content. The MBC was estimated using the fumigation–extraction method^57^. Fumigated and nonfumigated soils were extracted using 0.5 M K_2_SO_4_ for 30 min (soil: extractant ratio, 1:4), and organic carbon in the soil extracts was measured by oxidation with potassium dichromate and titration with ferrous ammonium sulfate. The MBC was calculated as *E*_*C*_/*K*_*EC*_, where *E*_*C*_ is the organic carbon extracted from the fumigated soil minus the organic carbon extracted from the nonfumigated soil, and *K*_*EC*_ is 0.38.

### Soil microbial community structure analysis

Soil microbial community structure was determined by PLFA analysis as described by Bossio *et al*.^58^. Lipids were extracted from 3 g of freeze-dried soil using a single-phase chloroform–methanol–citrate buffer (1:2:0.8). The soil extracts were filtered and the chloroform phases were collected. Polar lipids were separated from neutral and glycol lipids on solid-phase extraction columns (Supelco Inc., Bellefonte, PA, USA) by eluting with CHCl_3_, acetone, and methanol. Phospholipids were treated with a mild-alkali methanolysis, and the produced fatty acid methyl esters were subsequently extracted using hexane and dried in N_2_. Samples were analysed using an Agilent 7890 A series Gas Chromatograph equipped with MIDI peak identification software (Version 4.5; MIDI Inc., Newark, DE, USA). Before analysis, samples were dissolved in hexane that contained 19:0 as an internal standard. Fatty acids are described according to the nomenclature of He *et al*.^59^. PLFAs that contributed less than 0.5% of the total amount extracted from each sample were eliminated from the dataset; consequently, 29 PLFAs were included in the statistical analysis. The total microbial biomass in each sample was calculated by summing the molar concentrations of all of the PLFAs.

Total bacteria were identified using the following PLFAs: 15:0, 17:0, i15:0, i16:0, i17:0, a15:0, a17:0, 16:1ω7c, 18:1ω7c, cy17:0, and cy19:0^60–62^; G− bacteria were identified using the following PLFAs: 16:1ω5c, 16:1ω7c, 16:1ω9c, 17:1ω8c, 18:1ω5c, 18:1ω7c, 18:1ω9c, cy17:0, and cy19:0^61, 63^; and G+ bacteria were identified using the following PLFAs: i14:0, i15:0, i16:0, i17:0, a15:0, and a17:0^64, 65^. The PLFAs 18:2ω6,9c and 18:1ω9c were chosen to identify fungi^65, 66^, and 10Me16:0, 10Me17:0, and 10Me18:0 to identify actinomycetes^67, 68^. The ratio of fungal PLFAs (18:2ω6,9c and 18:1ω9c) to bacterial PLFAs was used to calculate the F/B ratio. The PLFAs 14:0, 15:1ω6c, 16:1ω7c, 16:1ω7t, 18:1ω9c, and 18:1ω9t were chosen to identify aerobic bacteria^52, 69, 70^; and i15:0, a15:0, i16:0, 10Me16:0, i17:0, a17:0, and 10Me18:0 to identify anaerobic bacteria^52, 71^; and 16:1ω5c to identify AMF. All of the PLFA biomarkers used were considered representative of the total PLFAs in the soil microbial community^2^.

### Statistical analysis

The data were analysed using SPSS software (version 16.0), and the treatment means were compared using the least significant difference test at *p* < 0.05. Pearson’s correlation analyses were conducted to investigate relationships between soil physicochemical properties and microbial parameters. Differences in soil microbial community composition were investigated using principal components analysis in Canoco for Windows (version 4.5).

### Data Availability

The datasets generated during and/or analysed during the current study are available from the corresponding author on reasonable request.
